# On Morbid Infection

**Published:** 1815-08

**Authors:** Robert Kinglake

**Affiliations:** Taunton


*For the London Medical and Physical Journal.*

### *On Morbid Infection*; by Dr. Kinglake.

THE various conditions of matter that may possess the power of inducing specific morbid effects on the animal economy, have not been traced out with sufficient precision to admit of any practical conclusions on the subject. The examples furnished by the different diseases that are directly and unquestionably infectious, evince that much of the same general nature may exist in other forms of disease, in which less distinctness of influence occurs. The plague, small-pox, measles, hooping-cough, typhus, scarlatina, syphilis, &c. exert infectious powers that leave no doubt of their efficacy in disseminating their specific influence. The infectious quality is, in those instances, so active as to overcome any resistance that may be opposed to its power, by either a constitutional or accidental state of irritability that might effectually counteract a less decided cause of infection. It is the absolute power of this high degree of morbid influence, that has almost exclusively drawn attention to it, as forming the basis of what has been held to be the real infectious nature of disease. When it must be considered, that a principle of influence is active in the animal economy, it becomes difficult to say how far it may not be operative under a variation of circumstances that may greatly modify and extend its agency without radically changing its character. If a diseased action, denominated variolous, should induce changes on the animal economy that would cause a similar disease to arise in any person who might come within the sphere of its activity, can it be truly affirmed, that other diseases do not, in a degree, also induce changes, which, though not equally certain in being propagated, yet, by possibility, may exert an infectious influence in circumstances favourable to its operation? All diseased actions are deviations from the healthful condition of vital power, and, in as far as some result must occur demonstrative of the morbid change, it is conceivable that such an effect might possess a quality capable of inducing a similar state of disease. The fixedness of health health opposes a salutary barrier to the inroad of slight morbid aggressions; but, when the resistance is less powerful, the noxiousness of the morbid power may gain a temporary ascendancy and develope itself in the specific character of the disease it may have excited. Happily for mankind, the various qualities of morbific matter are not sufficiently powerful to establish even a short-lived dominion over the tone and energy of health; but it is in the diminution of the healthful state, that the trembling powers of life become a prey to noxious power, and render diseases infectious, which, in more salutary circumstances, could not have exerted such an efficiency.

If, therefore, the principle of infection may be recognised in all deviations from the healthy state, although its active influence may depend on the existing condition of vital power, it may be justly held that absolute exemption from the spread of diseases of any description does not exist. Looking, therefore, through the heavy catalogue of morbid affections to which human nature is liable, it is impossible not to feel some well founded apprehension that each form of disease may, under certain circumstances, communicate and extend itself by a sort of *materies morbi*, occupying a smaller or larger sphere of infectious power, according to the quality and efficiency of its separate and component parts.

In advocating, or rather suggesting, the possibility of the change induced by the diseased state, whatever may be its degree and character, possessing a morbific quality capable of being communicated under circumstances favourable to its diffusion, nothing like precision or definitive conclusion can be offered on the subject. It is sufficient, as before observed, to know that certain forms of disease are unquestionably infectious, and that others of a more equivocal nature in that way do occasionally appear to be extended by an infectious quality. Of this description, may certainly be stated pulmonary consumption, and, perhaps, most forms of febrile affection. Cutaneous eruptions, and ill-conditioned ulcers, furnish exhalations imbued with a greater or less degree of infectious power.

The possibility of morbid actions, of whatever denomination, generating and evolving an arrangement of matter that might prove infectious, suggests the importance of duly regarding such precautions against its influence as may securely obviate its becoming injurious. For this purpose, free ventilation and the most scrupulous attention to personal and domestic cleanliness should be assiduously observed.

In

In the more evident instances of infectious power, much may be done in diminishing, and often in effectually counteracting, its influence; and an anxious endeavour should be exerted at all times, at least to lessen, if not wholly to prevent the prevalence of morbid infection. Frequent personal ablutions and effectual washings of the floors, utensils, and general furniture of the apartments of the diseased, should be enjoined and strictly executed. The vehicles of infection would then be destroyed, the focus of its power would be broken, wholesome air would occupy the room of noxiously impregnated vapour, and an happy exemption very generally would be enjoyed from every species of disease, whether regarded as of an infectious nature or not.

Some time since, I submitted to the public an intimation of my suspicion, that a decree of communicable infection existed in phthysis pulmonalis. This opinion was induced by the striking fact of one sister quickly following another to the grave through that destructive distemper, in neither of whom was there any thing either of hereditary or constitutional tendency to have produced the disease in the signal manner in which it appears to have been occasioned by the latter victim being almost incessantly associated with her sister during the progress of her mortal distemper. Since that time other instances have occurred, from which a similar inference might be drawn, but not in so unequivocal a manner as in the cases referred to.

The experienced medical practitioner must have in his recollection numerous instances of one near relative involving another in the deadly toils of phthysis pulmonalis, and that in the exact proportion in which the personal intercourse and association might have been more or less close during the destructive progress of the disease. The long duration of this baneful disease, the assiduity of personal attendance on the patient, the hectic state of the general system, the febrile exacerbations, the vitiated state of the secretions, especially that of the expectorated mucus, the anxious respiration, and the necessity for an earnest attendant often to sustain the patient in an elevated position, during which time respiration must be performed in the contaminated atmosphere of the lamented sufferer; these, and other sources of morbid infection are collectively powerful enough to make diseased impressions on the stoutest constitution, and more particularly on such weakly persons as may be considered as naturally predisposed to morbid affections.

This subject seems to have interested the attention of Dr. Haxby, from an observation lately made by him in one of

 your your numbers with reference to myself. The enquiry is certainly worthy of the general notice of the medical faculty; and, if Dr. Haxby should more particularly give it his mature consideration, I am persuaded, from my former knowledge of his studious habits and professional acquirements, that he would largely contribute towards elucidating the question and ascertaining what may be its real importance in medical practice. No opportunity that may present to me for further investigation of the subject shall be neglected, and whatever results may arise of a conclusive character, shall be freely submitted to public consideration, that the practical worth of the inquiry may be correctly appreciated and determined.

*Taunton;* ROBERT KINGLAKE.

*June* 21, 1815.

